# Function of ALA Content in Porphyrin Metabolism Regulation of *Ananas comosus* var. *bracteatus*

**DOI:** 10.3390/ijms24065274

**Published:** 2023-03-09

**Authors:** Mark Owusu Adjei, Jiaheng Luo, Xi Li, Juan Du, Aiping Luan, Shujiang Li, Jun Ma

**Affiliations:** 1 College of Landscape Architecture, Sichuan Agricultural University, Chengdu 611100, China; 2 College of Forestry, Sichuan Agricultural University, Chengdu 611130, China; 3 Tropical Crop Genetic Resources Institute, Chinese Academy of Agricultural Science, Haikou 571101, China

**Keywords:** chimeric leaf, 5-aminolevulinic acid, chlorophyll, heme, *hemA*

## Abstract

Chlorophyll and heme are essential molecules for photosynthesis and respiration, which are competing branches of the porphyrin metabolism pathway. Chlorophyll and heme balance regulation is very important for the growth and development of plants. The chimeric leaves of *Ananas comosus* var. *bracteatus* were composed of central photosynthetic tissue (PT) and marginal albino tissue (AT), which were ideal materials for the study of porphyrin metabolism mechanisms. In this study, the regulatory function of ALA content on porphyrin metabolism (chlorophyll and heme balance) was revealed by comparing PT and AT, 5-Aminolevulinic Acid (ALA) exogenous supply, and interference of *hemA* expression. The AT remained similar in porphyrin metabolism flow level to the PT by keeping an equal ALA content in both tissues, which was very important for the normal growth of the chimeric leaves. As the chlorophyll biosynthesis in AT was significantly inhibited, the porphyrin metabolism flow was directed more toward the heme branch. Both tissues had similar Mg^2+^ contents; however, Fe^2+^ content was significantly increased in the AT. The chlorophyll biosynthesis inhibition in the white tissue was not due to a lack of Mg^2+^ and ALA. A 1.5-fold increase in ALA content inhibited chlorophyll biosynthesis while promoting heme biosynthesis and hemA expression. The doubling of ALA content boosted chlorophyll biosynthesis while decreasing *hemA* expression and heme content. *HemA* expression interference resulted in a higher ALA content and a lower chlorophyll content, while the heme content remained at a relatively low and stable level. Conclusively, a certain amount of ALA was important for the stability of porphyrin metabolism and the normal growth of plants. The ALA content appears to be able to regulate chlorophyll and heme content by bidirectionally regulating porphyrin metabolism branch direction.

## 1. Introduction

*Ananas comosus* var. *bracteatus,* also known as a variegated pineapple, is a perennial evergreen herb that belongs to the genus Bromeliaceae [1]. It is planted as an ornamental plant due to its beautiful photosynthetic-albino chimeric leaf and red fruit. Its chimeric leaf is made up of central photosynthetic tissue (PT, which contains chlorophyll and is found in the center of the leaves) and marginal albino tissue (AT, which lacks chlorophyll and is found on both edges of the leaves) [2]. Over the years, studies on *Ananas comosus* var. *bracteatus* have revealed biological mechanisms that control the leaf type, and they are ideal research materials for further revealing plant growth, porphyrin metabolism, and development regulation mechanisms [3,4,5]. Leaf albinos are common leaf color mutants that exhibit an inability to produce chloroplasts as well as defects in chlorophyll biosynthesis. However, the albino tissues of chimeric leaves are different from the very weak albino mutations; they can grow well since they cooperate with the normal photosynthetic leaf tissue in growth and development. The porphyrin regulation mechanism of the chimeric leaves must be different from the albino mutant, and it is interesting to reveal the porphyrin metabolism regulation mechanism of the chimeric tissue types. There are enzymatic steps involved in porphyrin metabolism and synthesis [6]. These reactions are closely regulated to prevent phytotoxic accumulation and ensure the continuous supply of enzymatic capacity to cognate cells [7]. In plants, the reactions are coordinated by chlorophyll synthesis [8], mediated by retrograde signals from chloroplasts [9] and cell cellular metabolism processes [10]. Studies have shown that the albino of the leaf is closely related to chloroplast development [11], chlorophyll synthesis [12], and heme metabolism [13]. Leaf color mutation, also known as chlorophyll mutation, is directly or indirectly related to chlorophyll biosynthesis dynamics [14].

The crucial co-factors and pigments for chlorophyll, heme, and phytochromobilin are produced by the porphyrin biosynthesis pathway [14]. All living cells produce porphyrin through a process called biosynthesis, and the first committed intermediary in this process is ALA [15]. In most cases, protoporphyrinogen oxidase is the last enzyme before the branch in the porphyrin biosynthetic pathway catalyzes the oxidation of protophorinogen IX to Protoporphyrin IX (Proto IX). Protophorinogen IX is then directed to the magnesium (Mg) and iron (Fe) branches for chlorophyll and heme biosynthesis, respectively [16,17].

Chlorophyll and heme are essential molecules for photosynthesis and respiration, generated through porphyrin metabolism [18,19]. The chlorophyll photo-pigments are necessary for photosynthesis, while heme is the precursor of the phytochrome component for the photosynthetic electron transport chain b_6_/f complex [20,21]. As the first and key precursor of porphyrin metabolism, ALA is generated through three-step enzymatic reactions catalyzed by glutamyl tRNA reductase (GluTR), which is coded by the *hemA* gene [22]. The GluTR catalyzation steps involve the synthesis of ALA from glutamyl tRNA, which is the first step of chlorophyll synthesis and a key rate-limiting step that determines how fast the whole chlorophyll pathway can be carried out [23]. Moreover, ALA is catalyzed to form the Protoporphyrin Ⅸ complex that binds to Mg^2+^ or Fe^2+^ and then enters the chlorophyll synthesis pathway and the heme synthesis pathway, respectively [24]. In higher plants, GluTR is always maintained in a stable homeostasis to avoid the unnecessary synthesis of tetrapyrrole compounds. In rice, for example, an uncontrolled accumulation of porphyrins and magnesium porphyrins resulted in a high formation of reactive oxygen species [25]. It has been revealed in tomatoes that heme content has a negative feedback control on GluTR and ALA content, and consequently the chlorophyll content [26]. Furthermore, heme acts on approximately 30 amino acid residues of the N-terminus of GluTR1 proteins, inhibiting GluTR activity in *Arabidopsis thaliana* [27]. The *AtGluTR* protein was encoded by *AtHemA1*, which regulates the balance of chlorophyll and heme metabolism in *Arabidopsis thaliana* [28]. Heme is not only involved in the synthesis of oxide-reductase co-groups but also in protein stabilization [29]. In *Pakchoi*, high expression of *hemA* inhibited the synthesis of chlorophyll, resulting in lower chlorophyll content and yellowing leaves [30]. In *Arabidopsis thaliana*, the *AtHemA1* expression resulted in the decrease in ALA, chlorophyll, and heme contents and a significant decrease in GluTR enzyme activity [31,32]. The protein encoded by *HemA* influences the role of GluTR in controlling the balance of chlorophyll and heme biosynthesis [33]. This shows that the interaction mechanism between ALA content, *hemA* expression, and GluTR content is a key regulatory element of porphyrin metabolism.

The chimeric leaves of *Ananas comosus* var. *bracteatus* have effective photosynthesis ability and grow normally, which provide typical normal photosynthetic tissues and albino tissues for studies on the porphyrin metabolism internal regulation mechanism in normally growing plants. Research results from chimeric leaves can make up for the shortcoming of studying porphyrin metabolism using albino mutants that do not grow normally. In this study, a comprehensive analysis of chlorophyll and heme biosynthesis in the photosynthetic central tissue and marginal albino tissue of *Ananas comosus* var. *bracteatus* chimeric leaves was conducted to reveal the relationship and balance between the two main branches of porphyrin metabolism. Exogenous ALA supply and *hemA* gene expression interference were used to reveal the regulatory function of ALA content on chlorophyll and heme biosynthesis balance.

## 2. Results

### 2.1. Porphyrin Metabolism Characters in Chimeric Leaves

**Figure 1:**
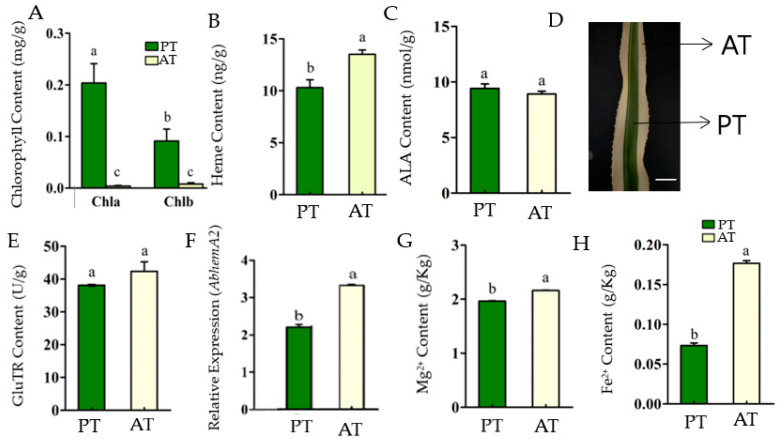
The porphyrin metabolism characterizes in the central photosynthetic tissue (PT) and marginal albino tissue (AT) of the chimeric leaves of *Ananas comosus* var. *bracteatus*. Chlorophyll content (**A**), heme content (**B**), 5-aminolevulinic acid content (**C**), Chimeric leaf (Photosynthesis tissue (PT) and marginal Albino tissue (AT) at 100 µm scale bar (**D**), GluTR content (**E**), Relative expression of *AbHemA2* (**F**), Mg^2+^ content (**G**), and Fe^2+^ content (**H**). The bars show how each bar differs from the others. The means denoted by the same letter did not differ significantly at *p* < 0.05.

Porphyrin metabolism provides the vital pigments for chlorophyll and heme [34]. Its biosynthesis begins with the formation of 5-aminolevulinic acid (ALA) as the first step [35]. The study examined the porphyrin metabolism characters in the PT and WT of *Ananas comosus* var. *bracteatus* chimeric leaves (Figure 1D). Unlike the albino mutation, chimeric leaves grow well, making them an ideal material for further research into the porphyrin metabolism mechanism.

The chlorophyll content of AT was significantly lower than that of PT (*p* < 0.01) (Figure 1A). However, the heme content in AT was significantly higher than that in PT (*p* < 0.05) (Figure 1B). Moreover, the heme to Chl ratio in AT was significantly higher than in PT. As the results suggest, the porphyrin metabolism flow in the AT is directed more to the heme branch than the chlorophyll branch, which resulted in the accumulation of heme and the lack of chlorophyll.

The rate-limiting step of porphyrin biosynthesis is ALA formation [36]. ALA is the first precursor of the chlorophyll synthesis pathway, synthesized by GluTR catalyzation. ALA plays a critical role in the chlorophyll biosynthesis pathway [37]. There was no significant difference in ALA content between PT and AT (Figure 1C). It was suggested that the inhibition of chlorophyll accumulation in AT did not result in the accumulation of ALA. The ratio of Chl/ALA in AT was lower than that of PT, while the Heme/ALA ratio was significantly higher than that of PT. Since the ALA content is basically the same between PT and AT, it was suggested that ALA in PT mainly flowed to the chlorophyll synthesis pathway, while AT mainly flowed to the heme synthesis pathway. 

It was revealed that ALA is generated through three-step enzymatic reactions catalyzed by GluTR, coded by the *hemA* gene [22]. There was no significant difference in GluTR content between PT and AT (Figure 1E), while the expression level of *AbHemA2* was significantly higher in AT than in PT (Figure 1F). It was indicated that both PT and AT maintained a similar specific level of porphyrin metabolism by maintaining a certain level of GluTR and ALA content. Additionally, the GluTR content was not only determined by *hemA* expression level. The translation and modification may affect the protein content as well. 

Generally in higher photosynthetic plants, the synthetic pathway of tetrapyrrole compounds is divided into two parts: ferrochelatase catalyzes the reaction insertion of Fe^2+^ into Proto IX to form Heme, and magnesium chelatase catalyzes the reaction insertion of Mg^2+^ into Proto IX to form Mg^2+^ protoporphyrin IX and then chlorophyll [24]. Moreover, Mg^2+^ is a component of chlorophyll involved in photosynthesis, and the synthesis of chlorophyll that requires the presence of Fe^2+^ [38]. Together, the Mg^2+^ and Fe^2+^ contents were closely related to the synthesis of chlorophyll and heme processes. In this study, the Mg^2+^ and Fe^2+^ contents in AT were significantly higher than those in PT (*p* < 0.05), especially the content of Fe^2+^, which was about 2.4 times to PT (Figure 1G,H). The presence of adequate levels of Mg^2+^ in AT suggested that Mg^2+^ content was not the limiting factor in AT chlorophyll biosynthesis. The Mg^2+^ content in AT may be promoted by the high concentrations of Fe^2+^ in it to maintain the specific level of ALA and the balance of porphyrin metabolism because Mg^2+^ and Fe^2+^ are co-factors that work in parallel [38,39]. The Mg^2+^ and Fe^2+^ contents were closely related to the synthesis of chlorophyll and heme processes. The Mg^2+^ and Fe^2+^ contents of AT were significantly higher than those of PT (*p* < 0.05), especially the content of Fe^2+^, which was about 2.4 times to PT (Figure 1F,G).

### 2.2. Effects of Exogenous ALA Supplementation on Porphyrin Metabolism

**Figure 2:**
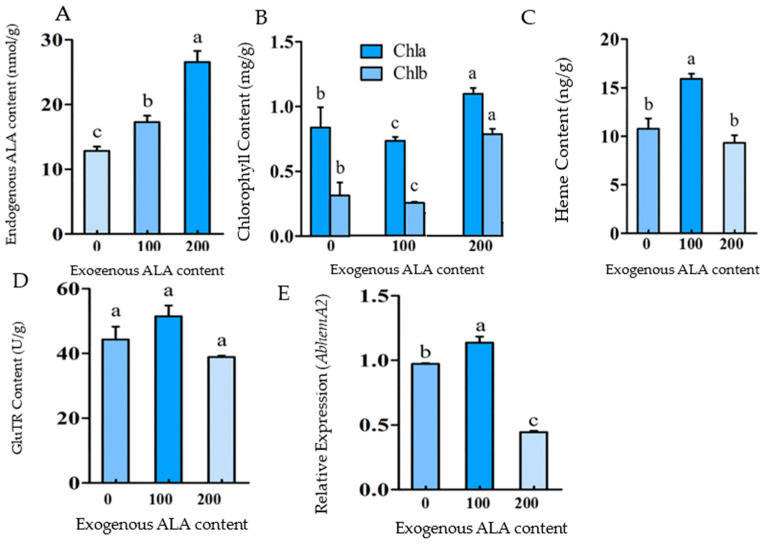
The effects of exogenous ALA supplementation on porphyrin metabolism. The effect of exogenous ALA supply on endogenous ALA content (**A**), The effect of exogenous ALA supply on chlorophyll content (**B**), The effect of exogenous ALA supply on heme content (**C**), The effect of exogenous ALA supply on GluTR content (**D**), and the effect of exogenous ALA supply on *AbHemA2* expression (**E**). The bars represent how each bar is different from the others. The means denoted by the same letter did not differ significantly at *p* < 0.05.

In order to study the effects of endogenous ALA content on porphyrin metabolism, exogenous ALA was supplied to increase the endogenous ALA content of the leaves. Different concentrations (0, 100, and 200 mg/L) of exogenous ALA were supplied to the photosynthetic leaves, and the endogenous ALA contents were detected after 2 days. After the treatment with exogenous ALA treatments of 100 mg/L and 200 mg/L, respectively, there was an increase in the endogenous ALA content in the leaves by approximately 1.4 and 2 times, respectively (Figure 2A). The results showed that exogenous ALA can effectively increase endogenous ALA levels, and that the higher the exogenous ALA concentration, the higher the endogenous ALA concentration.

The chlorophyll and heme contents were determined 2 days after the exogenous ALA treatment. The heme content of the 100 mg/L ALA treatment group significantly increased, which was about 1.5 times that of the control group (0 mg/L); however, the chlorophyll content decreased significantly (Figure 2B). It was proposed that 100 mg/L exogenous ALA (endogenous ALA content increased to about 1.5 times) promoted heme synthesis while inhibiting chlorophyll synthesis. When the exogenous ALA was increased to 200 mg/L, the heme content decreased with no significant differences from the control, but the chlorophyll content increased significantly (Figure 2C). It was indicated that 200 mg/L exogenous ALA (the endogenous ALA content increased to about 2 times) promoted the biosynthesis of chlorophyll. In conclusion, these results confirmed that ALA content can regulate the branch direction (chlorophyll or heme) of the porphyrin metabolism. A certain improvement of ALA content (about 1.5 times) can promote the biosynthesis of heme and inhibit the biosynthesis of chlorophyll, but an excessive enhancement of ALA (about 2 times) will promote the biosynthesis of chlorophyll and maintain heme biosynthesis on a certain level. 

In the balance regulation of chlorophyll and heme biosynthesis, ALA content plays an important bidirectional regulatory role. GluTR is the key enzyme in ALA biosynthesis and is encoded by the *HemA* gene. The increase in endogenous ALA content caused by exogenous ALA supply did not appear to affect GluTR content significantly (Figure 2D). It was discovered that the increase in ALA content caused by exogenous ALA supply did not result in GluTR feedback regulation. The relative expression of *AbHemA2* increased significantly under 100 mg/L ALA treatment and decreased significantly under 200 mg/L (Figure 2E). The exogenous-supply-induced ALA content increase can regulate the expression of the *AbHemA2* gene bidirectionally.

### 2.3. Effects of HemA Expression on Porphyllrin Metabolism

**Figure 3:**
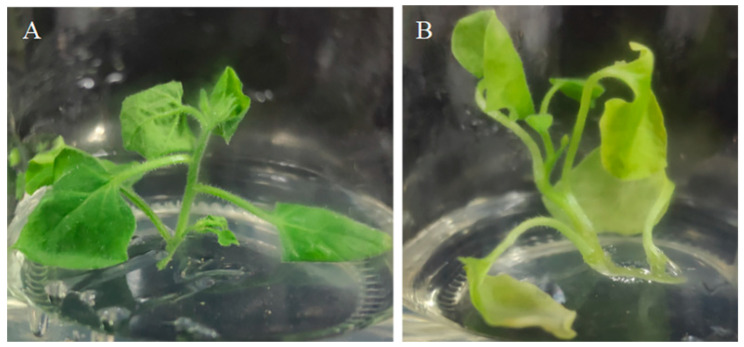
The wild type (**A**) and pTCK303-*AbHemA2*-RNAi transgenetic plants (**B**).

In order to reveal the effects of *hemA* gene expression on porphyrin metabolism, the conserved sequence fragment of the *hemA* gene between tobacco and *Ananas comosus* var. *bracteatus* was used to construct the interference vector (pTCK303-*AbHemA2*-RNAi) and transformed into tobacco. Compared to the wild-type plant, the interference plant was yellowish and some parts of the leaves even turned albino (Figure 3). 

**Figure 4:**
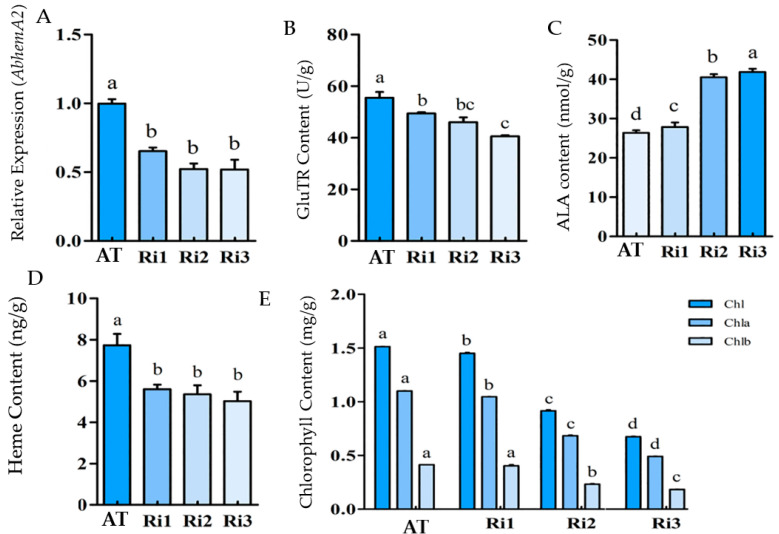
Effects of *HemA* expression interference on porphyrin metabolism. Relative *HemA* expression using *Tubulin* as the reference gene (**A**), GluTR content (**B**), ALA content (**C**), heme content (**D**) and chlorophyll content (**E**). The means denoted by the same letter did not differ significantly at *p* < 0.05.

The expression of *hemA* in the three transgenic tobacco lines (Ri1, Ri2, and Ri3) was significantly lower than the wild type (*p* < 0.05) (Figure 4A). It was confirmed that the expression of *hemA* was inhibited effectively. The GluTR content of the transgenic plants decreased significantly as *hemA* gene expression decreased (Figure 4B), indicating that *hemA* expression regulation affected GluTR content. It was worth noting that the ALA content of the transgenic plants increased significantly (about 1.5 times to that of the wild type) (Figure 4C), which was not in accordance with the decreasing *hemA* expression and GluTR content. However, the chlorophyll and heme content of the transgenic plants were significantly lower than that of the wild type (Figure 4D,E). The increased accumulation of ALA (<1.5 times) in the transgenic plants resulted in a greater decrease in chlorophyll biosynthesis, while the heme content remained at a relatively stable lower level.

## 3. Discussion

Chlorophyll and heme are the two main branches of the porphyrin metabolism pathway. The metabolism pathway begins with the first precursor, ALA, which is synthesized by the catalyzation of GluTR, and branches to heme or chlorophyll by the binding of protoporphyrin IX to Fe^2+^ or Mg^2+^ separately. Heme is an iron-containing cyclic tetrapyrrole compound and an intermediator in phytochrome and phycobilin synthesis [31]. Chlorophyll is synthesized by the magnesium branch of the porphyrin metabolism pathway and is very important for photosynthesis [40]. The synthesis of ALA is the key limitation step of chlorophyll and heme biosynthesis [41], and the content of heme has a negative feedback regulation on the content of ALA and GluTR content [13]. 

The central photosynthetic and marginal albino tissues of the chimeric leaves of *Ananas comosus* var. *bracteatus* cooperated to keep normal growth and development of the plants. Comparing the porphyrin metabolism characters between the photosynthetic and albino tissues of the chimeric leaves can better illustrate the internal cooperation mechanism of chlorophyll and heme biosynthesis in plants than using albino mutants as study material. The similar ALA and GluTR content of the photosynthetic and albino tissues of the chimeric leaves suggested that the two tissues kept a similar porphyrin metabolism level. Chlorophyll and heme are essential molecules for photosynthesis and respiration [18,19]. Maintaining a certain level of porphyrin metabolism in both photosynthetic and albino tissues is important for the normal growth of the chimeric leaves and plants. The albino tissues of the chimeric leaves stabilized porphyrin metabolism by increasing heme biosynthesis and compensating for the damage caused by chlorophyll biosynthesis inhibition. The accumulation of heme in the *Arabidopsis* mutation inhibited chlorophyll biosynthesis and resulted in leaf albinism [42]. The increased heme content in the albino tissue of *Ananas comosus* var. *bracteatus* chimeric leaves may give destructive feedback, regulating chlorophyll biosynthesis, resulting in the albino color of the leaves. 

The biosynthesis of chlorophyll and heme is branched by the ion binding of protoporphyrin IX. Ferrous chelatase catalyzes the insertion of Fe^2+^ into Proto-IX to form the ferrum branches of Heme and phytochromes, while magnesium chelatase catalyzes the insertion of Mg^2+^ into Proto-IX to form the magnesium branch of Mg-protoporphyrin IX. [43]. The availability of Fe^2+^ and Mg^2+^ will affect the biosynthesis of heme and chlorophyll. As a component of chlorophyll, Mg^2+^ is an essential element for plant growth and development. It can participate in photosynthesis, maintain the stability of the cell membrane, and regulate the enzymes activity in cells [44,45]. The albino tissues of the chimeric leaves contained significantly more Fe^2+^ and Mg^2+^ than the photosynthetic tissues, indicating that the inhibition of chlorophyll biosynthesis in the albino tissues of the chimeric leaves was not due to a lack of Mg^2+^. It was reported that the expression level and protein abundance decrease in chlorophyll-biosynthesis-related genes and chloroplast development inhibition played important roles in the albino of *Ananas comosus* var. *bracteatus* chimeric leaves [46]. 

Although the biosynthesis of chlorophyll was significantly inhibited, the porphyrin metabolism flow in the albino tissue was maintained at a similar level to that in the photosynthetic tissues. To avoid ALA accumulation, the metabolism flow branched more toward the ferrum branch to form heme. It was reported that, as a non-protein amino acid, ALA acts like a growth regulator and plant hormone [15]. Keeping a relatively stable GluTR and ALA content may be one of the reasons why the albino tissue of the chimeric leaves can grow normally. In order to further analyze the regulation function of ALA content on porphyrin metabolism, exogenous ALA was supplied to regulate the endogenous ALA content of the plants. It is reported that exogenous supply of ALA can increase chlorophyll biosynthesis and photosynthesis of plants [47]. In our study, with the increased supplies of exogenous ALA, the endogenous ALA content of the leaves increased accordingly. When the endogenous ALA content increased to about 1.5 times, the biosynthesis of chlorophyll was inhibited and heme biosynthesis was promoted, apparently. When the endogenous ALA content increased to about 2 times, the biosynthesis of chlorophyll was promoted and the biosynthesis of heme decreased to be similar to that of the control. These results suggested that the plants need to keep ALA content at a specific level to maintain a certain level of porphyrin metabolism and a balance of chlorophyll and heme biosynthesis for the normal growth and development of the plant. These results showed that the plants stabilized porphyrin metabolism by redirecting metabolism flow to heme biosynthesis when ALA content increased appropriately (about 1.5 times), while chlorophyll biosynthesis would be increased, apparently, and heme biosynthesis would be kept at a lower level if ALA content increased more (about 2 times). The results further indicated that plants could not accumulate excessive heme, and maintaining the metabolic balance of chlorophyll and heme was very important for the normal growth of plants. Endogenous ALA content can regulate the biosynthesis of chlorophyll and heme bidirectionally, which functions effectively in the regulation of chlorophyll and heme balance and porphyrin metabolism stability.

The biosynthesis of ALA is catalyzed by the GluTR content, which is encoded by the *hemA* gene. The exogenous supply of ALA for *Ananas comosus* var. *bracteatus* leaves increased endogenous ALA content, but it did not affect GluTR content, apparently. It was suggested that ALA content did not have a feedback regulation function on GluTR content. When *AtHemA1* gene expression was silenced, the contents of ALA, chlorophyll, and heme in leaves decreased to varying degrees, and the GluTR content also decreased significantly, which confirmed for the first time the role of *AtHemA1* in chlorophyll and heme synthesis in higher plants [48]. The interference with *hemA* gene expression resulted in a decrease in GluTR, heme, and chlorophyll content. It was suggested that the porphyrin metabolism level was down-regulated by *hemA* interference. The decrease in chlorophyll content was more apparent than that of heme content, which indicated that heme-lacking was more damage for the plants than chlorophyll-lacking. The ALA content of the interference plants increased significantly, reaching approximately 1.5 times that of the wild type. The accumulation of ALA under the interference of *hemA* gene expression may be caused by the simultaneous inhibition of chlorophyll and heme biosynthesis, and the ALA accumulation may further inhibit chlorophyll biosynthesis while maintaining heme content at a relatively low level. This result was similar to the result of exogenous ALA supply experiment in banana plants [36]. 

## 4. Materials and Methods

### 4.1. Plant Material

*Ananas comosus* var. *bracteatus* chimeric plants with photosynthetic-albino striped leaves were used to detect pigment, ALA, heme, Mg^2+^, Fe^2+^ and GluTR contents, and *hemA* expression. Three biological replicates were set up for each sample.

### 4.2. Determination of Photosynthetic Pigments

The central photosynthetic and marginal albino tissues of the fresh and mature functional chimeric leaves were cut separately for the detection of chlorophyll a (Chl a), chlorophyll b (Chl b) and carotenoid (Car) contents [49]. About 0.1 g of each tissue was cut into 2 × 2 mm squares and soaked in 5 mL of 95% ethanol in the dark. The photosynthetic pigment isolation and measuring process were executed accordingly [4].

### 4.3. Determination of ALA Content

The ALA content was measured with some modifications [50]. The leaf tissues (0.5 g) were homogenized in 5 mL of trichloroacetic acid (*w*/*v* = 4%) and centrifuged at 10,000× *g* for 10 min. The assay mixture consisted of 10 mL of two combined supernatant, 5.7 mL of sodium acetate, and 0.15 mL of acetylacetone (pH 4.6). The assay medium was mixed and heated in a boiling water bath for 10 min. The extract was cooled to room temperature, and an equal volume of modified Ehrlich’s reagent was added (1:1), vortexed for 2 min, and incubated in the dark for 15 min. The extraction absorbance was measured at a wavelength of 553 nm, and the ALA standard curve was determined as described.

### 4.4. Determination of Heme Content

Fresh leaf samples (0.1 g each) were homogenized in motor with pistil in 0.9 mL of Phosphate Buffer Saline (PBS), pH 7.4, transferred to a 2 mL centrifuge tube, and centrifuged at 3000 rpm at 4 °C for 20 min. The supernatant was used for the Heme concentration assay. Heme content was determined using a Plant (Heme) ELISA Kit [Shanghai, China, mlbio.cn] and the content was measured with a microplate reader Model 680 (Bio-Rad, Hercules, CA, USA) according to the manufacturer’s instructions (Supplementary Materials) as described [51].

### 4.5. Determination of Mg^2+^ and Fe^2+^ Content

The central photosynthetic and marginal albino tissues of the fresh chimeric leaves were cut separately and oven dried. To 100 mL of a graduated digestion tube containing 15 mL of nitric acid and perchloric acid, mixed at a 4:1 ratio, 0.3 g of the tissue samples were added and allowed to soak overnight. At low and high temperatures, the samples were digested at 160 °C and 300 °C for about 1–2 h, respectively, and then cooled to room temperature. After sampling, according to the content type, the iron (Fe^2+^) and magnesium ion (Mg^2+^) standard curves were determined accordingly [40]. The absorbance was measured using an atomic absorption spectrophotometer at wavelengths less than 450 nm. 

### 4.6. Determination of GluTR Content

The GluTR content was determined using a Plant GluTR ELISA Kit, (Shanghai, China, mlbio.cn). The reference GluTR protein was diluted gradiently, and a standard curve was first established using the double-antibody sandwich enzyme-linked immunosorbent assay [52]. Fresh leaf samples (0.1 g) were homogenized in 0.9 mL PBS (pH 7.4) at 4 °C. The samples were centrifuged at 3000 rpm for 20 min, and the supernatant was used for the assay. The GluTR content (U/g) was calculated by a formula according to the standard curve [23]. Each sample was triplicated according to the manufacturer’s instructions (Supplementary Materials). 

### 4.7. Supply of Exogenous ALA

Plants regenerated from *Ananas comosus* var. *bracteatus* stem by tissue culture [4] were used for the ALA exogenous supply experiments. The tissue culture-regenerated plants were sprayed with exogenous ALA solutions at 0 mg/L, 100 mg/L, and 200 mg/L overnight and kept in the dark. Each treatment was repeated three times and arranged randomly. After 2 days, fresh leaves were collected, cleaned, quickly frozen in liquid nitrogen, and stored at −80 °C for subsequent index determination.

### 4.8. Expression Interference of hemA Gene

The conserved sequence of *hemA* gene family of *AbHemA2* was used for the construction of pTCK303*-AbHemA2-RNAi*. The vector was transformed into tobacco mediated by *Agrobacterium tumefaciens*. The expression analysis of *AbHemA2* in the wild-type and transformed plants was performed by qRT-PCR using *Tubulin* as the reference gene.

### 4.9. Data Analysis

The analysis of variance (ANOVA) in SPSS 20.0 Version was used to analyze all of the experiment data, with *p* ≤ 0.05 probability significant values. The pigment content determination data were calculated and plotted using SPSS, and Graph Pad Prism 5.

## 5. Conclusions

The regulation functions of ALA content in *Ananas comosus* var. *bracteatus* porphyrin metabolism were demonstrated in this study by comparing the photosynthetic and albino parts of the chimeric leaves, exogenous ALA supply, and *hemA* expression interference. The albino tissue stabilized porphyrin metabolism levels by maintaining a certain ALA content and directing the metabolism flow more to the heme branch to avoid excessive accumulation of ALA. The increase in heme content in the albino tissue may negatively regulate chlorophyll biosynthesis, resulting in albino chimeric leaves. The inhibition of chlorophyll biosynthesis in albino tissue was not caused by a lack of Mg^2+^ and ALA. A stable level of ALA content is very important for the normal growth of the chimeric leaves. Endogenous ALA content increased as a result of exogenous ALA supply and interference of *hemA* expression. The ALA content did not have a feedback regulation function on the GluTR content, but it apparently regulated chlorophyll and heme biosynthesis bidirectionally. Appropriate increases in ALA content inhibit chlorophyll biosynthesis and redirect metabolism flow to heme biosynthesis, whereas excessive increases in ALA content promoted metabolism flow to chlorophyll biosynthesis and maintained heme content at a normal level.

## Data Availability

Not available.

## Supplementary Materials

The following supporting information can be downloaded at: https://www.mdpi.com/article/10.3390/ijms24065274/s1.
